# Structural basis of transglucosylation in dextran dextrinase, a homolog of anomer-inverting GH15 glucoside hydrolases

**DOI:** 10.1016/j.jbc.2025.110541

**Published:** 2025-07-30

**Authors:** Takayoshi Tagami, Wataru Saburi, Juri Sadahiro, Yuya Kumagai, Weeranuch Lang, Naohiro Matsugaki, Masayuki Okuyama, Haruhide Mori, Atsuo Kimura

**Affiliations:** 1 Research Faculty of Agriculture, Hokkaido University, Sapporo, Japan; 2 Faculty of Fisheries Sciences, Hokkaido University, Hakodate, Japan; 3 Structural Biology Research Center, Photon Factory, Institute of Materials Structure Science, High Energy Accelerator Research Organization, Tsukuba, Japan

**Keywords:** carbohydrate biosynthesis, crystal structure, enzyme kinetics, enzyme structure, glycoside hydrolase, glycosyltransferase

## Abstract

Bacterial exopolysaccharide, dextran, primarily composed of α-(1→6)-linked d-glucosyl residues, is synthesized from α-(1→4)-glucan dextrin or sucrose through successive anomer-retaining transglucosylation reactions by dextran dextrinase (DDase) or dextransucrase, respectively. Although the structure–function relationship of dextransucrase has been extensively studied, that of DDase remains largely unknown. Herein, we revealed the *Gluconobacter oxydans* DDase structural basis through biochemical and structural analyses. The DDase comprises 1284 residues, with its N-terminal 902 residues being functionally essential. Crystal structure analysis of the minimal active DDase (Δ382C) complex with the pseudo-maltotetraose inhibitor, acarbose, revealed its homodimeric structure. A Δ382C protomer contains two β-sandwich domains, N1 and N2, and an (α/α)_6_-barrel domain A. Surprisingly, domains N2, A, and the helix-loop-helix connecting them structurally resemble those of bacterial anomer-inverting glucohydrolases in glycoside hydrolase family 15 (GH15). Domain N1 primarily forms intra- and inter-subunit domain interfaces. The DDase acarbose-binding residues in subsite −1 are conserved with GH15 glucohydrolases. The DDase Glu671 and Glu858 are positioned similarly to the GH15 glucohydrolase general acid and base catalysts, respectively. However, Glu858 is approximately 1.2 to 1.6 Å closer to the acarbose equivalent anomeric carbon, facilitating its role as a nucleophilic catalyst in the double displacement mechanism. The catalytic residue functions were biochemically confirmed using mutant enzymes. Spatial position of Glu858 is arranged by the local structure of the α11→α12 loop and subunit interactions involving domain N1. Enzymes classified in the same GH family catalyze reactions with different mechanisms, anomer-inverting or -retaining, due to differences in their catalytic residue spatial arrangement.

Dextran, primarily composed of α-(1→6)-linked d-glucosyl residues, is an exopolysaccharide produced by lactic acid and acetic acid bacteria and is widely utilized in food and pharmaceutical industries (1). This polysaccharide is synthesized through successive transglucosylation reactions catalyzed by transglucosidases: dextransucrase (DSase; EC 2.4.1.5) and dextran dextrinase (DDase; EC 2.4.1.2). DSase utilizes sucrose as the d-glucosyl donor substrate to produce dextran and is applied in the industrial dextran production (2). Conversely, DDase uses α-(1→4)-glucans such as starch hydrolysates, dextrins, and maltooligosaccharides (MOSs) as the d-glucosyl donor (3). DSase uses over 80% of the d-glucosyl residues of sucrose to produce dextran (4); however, the theoretical maximum yield of dextran based on sucrose amount is limited to 47% (w/w) because the d-fructosyl residues are not utilized. In contrast, DDase efficiently utilizes maltodextrin as a d-glucosyl donor, resulting in a higher yield of dextran, ranging from 58 to 74% (w/w) from short-chain amylose (5, 6).

DDase is produced only by particular strains of the acetic acid bacterium *Gluconobacter oxydans* (formerly known as *Acetobacter capsulatum* and *A. viscosus*), which is a causative agent of ropy beer (7). Two forms of DDase, one located extracellularly and the other on the *G. oxydans* cell surface, are identical (8). The relative quantities of these two forms change drastically during culture, influenced by the d-glucose and presence of MOSs (6, 7, 8, 9, 10). DDase catalyzes primarily three d-glucosyl transfer reactions: α-(1→4)-transglucosylation for MOSs resulting in their disproportionation, and α-(1→6)-transglucosylation for MOSs and isomaltooligosaccharides (IMOs) (11). It utilizes various oligosaccharides and glycosides possessing d-glucosyl or d-xylosyl residues at their non-reducing termini as acceptor substrates in transglucosylation (12, 13). As a consequence of the reactions, DDase converts MOSs into IMOs, isomaltomegalosaccharides, and dextran, which are α-(1→6)-linked carbohydrates consisting of 2 to 9, 10 to 100 (or up to 200), and over 100 to 200 days-glucose units, respectively (10, 14). Isomaltomegalosaccharides, which are abundantly produced by the DDase reaction, were found to exhibit distinctive and valuable properties, including slow digestibility and increasing the solubility of azobenzene dyes and aromatic drugs (15, 16, 17, 18).

Transglycosidase reactions follow the mechanism of retaining glycoside hydrolases. Glycoside hydrolases are classified based on their stereochemical outcomes: inverting and retaining enzymes, which produce reaction products with inverted or retained substrate anomeric configurations, respectively. Inverting enzymes follow the single-displacement mechanism involving general acid and base catalysts, whereas retaining enzymes follow the double-displacement mechanism involving general acid/base and nucleophile catalysts (19, 20). In the single-displacement reaction mechanism, the enzyme forms a ternary complex with carbohydrate and water. The general acid catalyst protonates the glycosidic oxygen, and the general base catalyst activates the water molecule for nucleophilic attack on the anomeric carbon from the opposite side of the glycosidic oxygen. In the double-displacement mechanism, a nucleophile catalyst attacks the anomeric carbon to cleave the glycosidic bond with the help of a general acid/base catalyst and forms a covalently bound glycosyl-enzyme intermediate. Subsequently, an acceptor molecule is deprotonated by the acid/base catalyst and attacks this intermediate to yield the product. Glycosyl acceptors are water in hydrolysis and carbohydrates and other molecules in transglycosylation.

Glycoside hydrolases are classified into 191 glycoside hydrolase families (GHs) in the CAZy database based on their amino acid sequence similarities (21). Members within the same family generally share a catalytic domain architecture and the catalytic mechanism. Exceptionally, GH23 and GH97 contain both inverting and retaining enzymes that possess the same residue serving as the proton donor (general acid and acid/base catalysts, respectively) but different residues as the other catalyst (general base and nucleophile, respectively) (22, 23, 24). GH15, one of the first GH families listed in the CAZy database (25), comprises inverting α-glucoside hydrolases: glucoamylase (EC 3.2.1.3) (26, 27), glucodextranase (EC 3.2.1.70) (28), α,α-trehalase (EC 3.2.1.28) (29, 30), and isomaltose glucohydrolase (EC 3.2.1.205) (31). These enzymes specifically hydrolyze the non-reducing terminal α-glucosidic linkage of MOSs, IMOs, trehalose (d-glucosyl (α1↔︎1α)d-glucoside), and isomaltose (IG2), respectively, and liberate β-d-glucose. GH15 enzymes share an (α/α)_6_-barrel catalytic domain and two catalytic glutamic acid (Glu) residues that function as the general acid and general base catalysts located in the α5→α6 and α11→α12 loops, respectively. Although GH15 has been regarded as a typical inverting glucohydrolase family, the synthesis of IMOs from MOSs by the C-terminal GH15 domain of the multi-domain protein, cycloisomaltooligosaccharide glucanotransferase (CITase) from *Thermoanaerobacter thermocopriae* (TtCITase) suggests the presence of retaining transglucosidases within GH15 (32).

DSase belongs to GH70, and its molecular structure has been well described (33, 34). The catalytic domain of DSase is an α-amylase-like (β/α)_8_-barrel that is circularly permutated compared to the catalytic domains of GH13 and GH77 enzymes, which form the GH-H superfamily with GH70 enzymes. DDase has been known since the 1950s, and several studies have investigated its enzymatic activity, including its application in carbohydrate production; however, its structural information has been lacking. This study demonstrates the structural similarity of DDase to GH15 inverting glucohydrolases by determining the DDase primary and three-dimensional structures. Furthermore, we discuss the molecular mechanism distinguishing DDase from GH15 inverting enzymes based on the DDase crystal structure and the analysis of its catalytic residue mutants.

## Results

### Sequence analysis of the DDase protein and encoding gene

Using the partial amino acid sequence of the native DDase purified from *G. oxydans* culture supernatant, the DDase gene and its flanking regions were obtained by gene cloning from the *G. oxydans* genomic DNA library and subsequent nested PCRs (Table S1 and Fig. S1). This resulted in identifying a 10,180 bp DNA sequence (GenBank accession number, LC008541) containing the 3855 bp DDase encoding gene (BAS29771.1) and four putative genes (ORF1–4). A BLAST search (35) suggested that ORF1, ORF2, and ORF3/ORF4 encode a glycosyltransferase, a haloacid dehalogenase-like hydrolase, and IS5 transposases, respectively, none of which are directly involved in dextrin or dextran metabolism. The DDase gene encodes a 1284-amino-acid protein. The deduced amino acid sequence contained all the partial sequences determined in the native *G. oxydans* DDase. The N-terminal amino acid sequence of the native DDase corresponds to Ala2–Ala11 of the deduced DDase sequence, indicating that DDase has no typical N-terminal secretion signal sequence.

**Figure 1:**
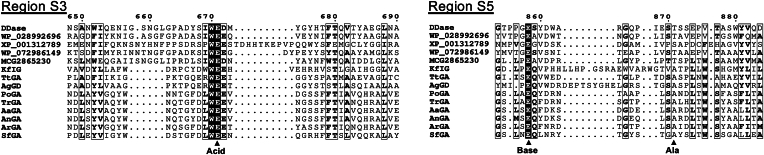
**Multiple sequence and structure alignment of regions S3 and S5 conserved in GH15 enzymes.** Multiple sequence and structural alignment of GH15 enzymes to compare the conserved regions S3 and S5 were constructed using PROMALS3D (80) and visualized using ESPript3 (81). The numerals indicate the amino acid sequence number of DDase. Acid, Base, and Ala indicate the general acid and base catalysts and a conserved Ala residue (Ala358 of KfIG, Ala649 of TtGA, and Ala650 of AgGD) among the inverting enzymes. Enzyme abbreviations with the corresponding PDB IDs are as follows: DDase, *Gluconobacter oxydans* dextran dextrinase (9JU0); KfIG, *Kribbella flavida* isomaltose glucohydrolase (5Z3A); TtGA, *Thermoanaerobacterium thermosaccharolyticum* glucoamylase (1LF6); AgGD, *Arthrobacter globiformis* glucodextranase (1ULV); PoGA, *Penicillium oxalicum* glucoamylase (6FHV); TrGA, *Trichoderma reesei* glucoamylase (2VN4); AaGA, *Aspergillus awamori* glucoamylase (1GAI); AnGA, *Aspergillus niger* glucoamylase (3EQA); ArGA, *Amorphotheca resinae* glucoamylase (6FHW); SfGA, *Saccharomycopsis fibuligera* glucoamylase (2FBA). The structures of DDase-similar proteins derived from *Thermoanaerobacter thermocopriae* (the C-terminal region of GH66 cycloisomaltooligosaccharide glucanotransferase, WP_028992696), *Trichomonas vaginalis* G3 (XP_001312789), *Clostridium cavendishii* (WP_072986149), and *Vulcanisaeta* sp. (MCG2865230) were predicted using AlphaFold3.

A BLASTP search of the DDase-deduced amino-acid sequence showed that putative homologous proteins with 97.4 to 98.8% sequence identity were present in *Gluconobacter vitians* (NCBI reference sequence number: WP_194260258.1), *Gluconobacter cerevisiae* (WP_194255978.1), and *Acetobacter malorum* (WP_061499504.1). The C-terminal GH15-like region of TtCITase (1184–1559; WP_028992696.1) showed 44% identity with the corresponding region of DDase. A DELTA-BLAST search (36) (threshold = 0.05) revealed sequence similarity of DDase to many GH15 glucohydrolases, such as *Thermoplasma volcanium* trehalase (30) (Uniprot ID: Q978S7; identity: 15%), *Aspergillus oryzae* glucoamylase (37) (Uniprot ID: P36914; identity: 11%), and *Kribbella flavida* NBRC 14399 isomaltose glucohydrolase (31) (KfIG; Uniprot ID: D2PPM8; identity: 17%). Multiple sequence alignment demonstrated that the general acid and base catalysts in the conserved regions of GH15 inverting glucohydrolases (26), S3 and S5, respectively, were conserved in DDase as Glu671 and Glu858, respectively, and in DDase-similar proteins, including the GH15 domain of TtCITase (Fig. 1). Other conserved regions, S1, S2, and S4, were also found in DDase (Fig. S2). AlphaFold3 (38) predicted the DDase four-domain structure: two N-terminal β-sandwich domains (Met1–Ala149 and Ser161–Ala422), the GH15-type (α/α)_6_-barrel catalytic domain (Ala503–Thr889), and a C-terminal β-helix domain (Gly922–Ala1284) (Fig. S3).

### Characterization of recombinant DDase and its truncated variants

Wild-type DDase was produced as a recombinant protein with an N-terminal His_6_ tag in *Escherichia coli* transformant and purified from its lysate *via* Ni^2+^-affinity column chromatography. Purified DDase showed a single band at 180 kDa on SDS-PAGE (Fig. S4). The molecular mass was similar to that of the native enzyme (Fig. S4), although it deviated from the theoretical mass of 136 kDa. The optimal pH and temperature for the d-glucose-releasing velocity from 200 mM maltose (G2) were pH 4.2 and 35 °C, respectively. The enzyme was stable at pH 2.2 to 6.0 (at 4 °C for 24 h) and at ≤ 40 °C (at pH 4.5 for 10 min). These enzymatic properties are similar to those of native DDase (5, 8, 39). During the reaction with maltotetraose (G4), the initial products were maltotriose (G3) and maltopentaose (G5) (Fig. S5). DDase predominantly catalyzed the α-(1→4)-d-glucosyl transfer reaction for a series of MOSs in the early reaction stage. The initial reaction rates on the MOSs followed the Michaelis–Menten equation, with the parameters listed in Table 1. The lowest *K*_m_ (7.8 ± 0.8 mM) and the highest *k*_cat_/*K*_m_ (42 s^−1^ mM^−1^) were obtained for G4 among the MOSs.

**Table 1:** Kinetic parameters of wild type, Δ382C, and E671Q

Substrate	Wilde type			Δ382C			E671Q		
	*K*_m_ (mM)	*k*_cat_ (s^−1^)	*k*_cat_/*K*_m_ (s^−1^ mM^−1^)	*K*_m_ (mM)	*k*_cat_ (s^−1^)	*k*_cat_/*K*_m_ (s^−1^ mM^−1^)	*K*_m_ (mM)	*k*_cat_ (s^−1^)	*k*_cat_/*K*_m_ (s^−1^ mM^−1^)
Maltose	130 ± 30	4.2 ± 0.5	0.032	160 ± 10	3.1 ± 0.3	0.019	–	–	–
Maltotriose	21 ± 1	470 ± 20	22	28 ± 3	190 ± 10	6.8	–	–	–
Maltotetraose	7.8 ± 0.8	330 ± 30	42	11 ± 2	150 ± 20	14	–	–	–
Maltopentaose	29 ± 4	360 ± 60	12	29 ± 1	160 ± 20	5.5	–	–	–
Maltohexaose	35 ± 2	350 ± 40	10	37 ± 6	140 ± 10	3.8	–	–	–
Maltoheptaose	16 ± 5	340 ± 30	21	31 ± 4	170 ± 10	5.5	–	–	–
Maltotetraose^a^	–	–	–	6.2 ± 0.1	140 ± 10	22	10 ± 1	0.0014 ± 0.0001	0.00014
α-d-Glucosyl fluoride^a^	–	–	–	40 ± 4	15 ± 1	0.38	1.5 ± 0.1	0.025 ± 0.002	0.017

The values were expressed as mean ± standard deviation of triplicate measurements.

The endashes indicate the values which were not determined.

a Rates measured in 130 mM sodium acetate buffer (pH 4.2).

**Figure 2:**
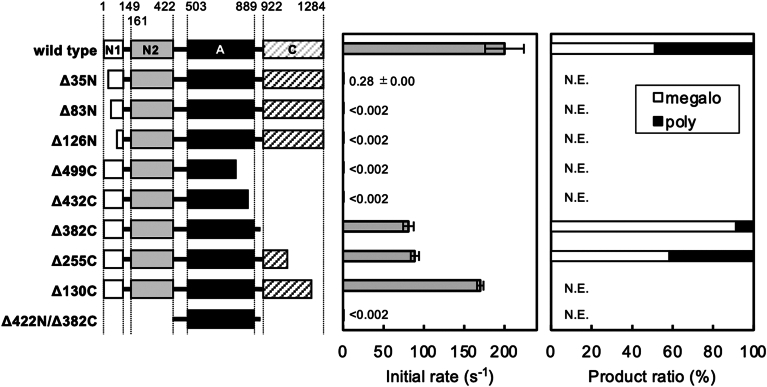
**Characterization of the truncated DDase variants.***Left*, schematic representations of the truncated DDase variants. The numerals indicate the residue numbers of DDase. *Center*, initial rates of G3 release from 15 mM G4 by the DDase variants. The values are presented as the mean with standard deviation of triplicate measurements. *Right*, weight distribution ratios of megalosaccharide (*white*) and polysaccharide (*black*), produced from maltooligosaccharides by the truncation variants. N.E.: not examined.

N- and C-terminally truncated forms of DDase were prepared to determine the regions essential for its activity (Fig. 2 and S4). Truncating the N-terminus resulted in a significant activity loss. A Δ35N mutant, lacking 35 residues from the N-terminus, showed only 0.14% wild-type DDase activity, and the further N-terminal truncation resulted in complete activity loss, even though the DDase variant ultraviolet circular dichroism spectra were similar to that of the wild type (Fig. S6). Conversely, C-terminal truncation mutants, Δ130C, Δ255C, and Δ382C, retained 41 to 85% wild-type activity, whereas more extensive truncations from the C- or N-termini (Δ432C, Δ499C, Δ422N/382C) showed no detectable activity. Therefore, the DDase minimal active form was determined to be Δ382C (Met1–Ser902), composed of domains N1, N2, and A. The wild-type and the active mutant Δ255C enzymes produced almost equal amounts of megalosaccharide (DP 10–200) and polysaccharide (DP > 100–200) from MOS, which were fractionated from the reaction products *via* a two-step precipitation with 40% and 90% (v/v) methanol. However, Δ382C produced much less polysaccharide than megalosaccharide. Δ382C showed similar enzymatic activities to those of the wild-type. The optimal pH and temperature for activity were pH 4.2 and 30 °C, respectively. The enzyme was stable at pH 2.2 to 6.0 (4 °C for 24 h) and at ≤ 37 °C (pH 4.5 for 10 min). Δ382C exhibited similar *K*_m_ values and 40 to 50% *k*_cat_ values for MOSs, compared with wild-type. The highest *k*_cat_/*K*_m_ (14 s^−1^ mM^−1^) was for G4, as observed in wild-type (Table 1).

### Crystal structure analysis of DDase-Δ382C

No crystal of the intact wild-type DDase was obtained under the tested conditions, whereas some co-crystals of Δ382C with acarbose were obtained. Acarbose is a pseudo-maltotetraose produced by *Actinoplanes* sp. and an effective inhibitor for exo-type α-glucoside-active enzymes such as glucoamylase, glucodextranase, and DSase (40, 41, 42). The co-crystal structure was solved by single-wavelength anomalous dispersion with sulfur atoms (S-SAD). The truncated mutant contains 20 Met and no Cys residues. Datasets (360° × 55) from a solvent-removed crystal were collected at a wavelength of 2.7 Å under helium atmosphere, and their merged data produced successful substructures with 66 anomalous peaks (occupancy > 0.3) (Table S2 and Fig. S7). A preliminary model at 3.1 Å resolution was built and used as a search model for the molecular replacement. Finally, the acarbose-bound Δ382C crystal structure was determined at a higher resolution, 2.5 Å.

**Figure 3:**
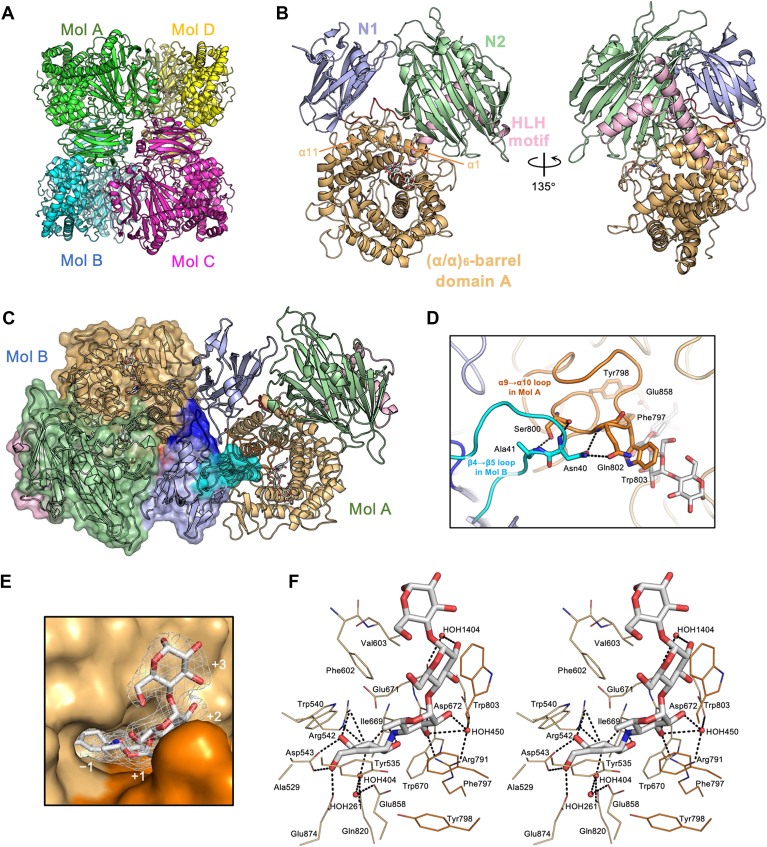
**Crystal structure of DDase-Δ382C in complex with acarbose.***A*, Overall structure of DDase-Δ382C. Each asymmetric unit contains two dimers (Mol A&B and Mol C&D). *B*, Domain organization of Δ382C. Δ382C is composed of N1 domain (Glu7–Ala149, *blue*), and N2 domain (Ser161–Ala422, *green*) and catalytic (α/α)_6_-barrel domain A (Ala503–Thr889, *orange*). Domains N2 and A are connected by a helix-loop-helix (HLH) motif (Thr423–Ser502, *pink*). The linker connecting the N1 and N2 domains is colored *red*. The acarbose bound to the active site is represented by a stick model. The α-helices 1 and 11 of the catalytic domain, which contact the N2 and N1 domains, respectively. (*C* and *D*) Overall and close-up view of dimer structure (Mol A&B). The color scheme is the same as in (*B*), but the β4→β5 (*cyan*) and β10→α1 (*navy blue*) loops of the N1 domain in Mol B (shown with surface model in (*C*)) and the α9→α10 loop (*red orange*) of domain A in Mol A are shown. Hydrogen bonds are shown as *dashed lines*. *E*, electron density (polder omit map contoured at 4 σ, *white* mesh) observed in domain A of Mol A. The subsite numbers (−1 to +3) are indicated. (*F*) Stereo diagram of the active-site structure of Mol A. Water molecules are shown as *red* spheres.

The crystal structure contains four molecules (Mol A–D) in an asymmetric unit (Fig. 3*A*). The region from Glu7 to Ser903 (Arg904 in Mol B) was modeled for each protomer. A loop region, Thr442–Ser445, was disordered in Mol B, C, and D. Proteins, Interfaces, Structure and Assemblies (PISA) (43) proposed that the four molecules in the asymmetric unit form two dimers (Mol A&B and C&D). The dimers Mol A&B and C&D shared almost identical structures, with a root mean square deviation of 0.255 Å over 120,759 atoms. The Δ382C molecular weight was estimated as 224,000 by gel filtration (Fig. S8), consistent with the theoretical dimer mass, 195,604 Da.

The Δ382C protomer overall structure comprised three domains: N1 (Glu7–Ala149), N2 (Ser161–Ala422), and catalytic (α/α)_6_-barrel domain A (Ala503–Thr889) (Figs. 3*B* and S9). Domains N2 and A were connected by a helix-loop-helix (HLH) motif (Thr423–Ser502) in which two α-helices are oriented perpendicularly. This domain organization was consistent with the structure predicted by AlphaFold3 with a difference in the N1 domain spatial position.

Domain N1 folded into an 11-stranded β-sandwich with a 3_10_-helix and an α-helix inserted at the β1→β2 and β10→β11 loops, respectively. Structural comparison using the DALI server (44) showed this domain is highly similar to the carbohydrate-binding module family 35 (CBM35) domain of GH66 *Bacillus circulans* T-3040 CITase (PDB ID: 3WNK; Z score, 15.2; RMSD, 1.8; align, 117 residues; sequence identity, 23%). The N-terminal truncation mutants Δ35N, Δ83N, and Δ126N, which exhibited very low or no enzymatic activity, lacked the regions from the N-terminus to β-strand 4, β-strand 7, and β-strand 10, respectively (Fig. S9).

Domain N2 is folded into an 18-stranded β-sandwich structure. A forty-five-residue β14→β15 loop contains two 3_10_ helices and an α-helix. This domain shares almost the same topology as the N-terminal domains of bacterial GH15 glucohydrolases, such as *Arthrobacter globiformis* I42 glucodextranase (AgGD; PDB ID: 1ULV) (28) and *Thermoanaerobacterium thermosaccharolyticum* ATCC7956 glucoamylase (TtGA; PDB ID: 1LF9) (45). These enzymes also contain an HLH motif that connects the N-terminal and catalytic domains.

The domain A (α/α)_6_-barrel structure is similar to GH15 inverting glucohydrolases. This domain does not contain extra secondary structures except for two short β-strands in the α7→α8 loop. Catalytic domain A contacts domains N1 and N2 within the same protomer *via* its α-helix 11 and α-helix 1 with their subsequent loops, respectively. Catalytic domain A is also in contact with domain N1 of the other protomer in the dimeric state at the domain A α9→α10 loop (Thr783–Trp818) and the domain N1 β4→β5 loop (Met30–Ser44) *via* hydrogen bonds (Fig. 3, *C* and *D*). Furthermore, the major dimer interface includes the domain N1 β10→α1 loop (Asn117–Pro130), which contacts each other.

### Active-site structure of DDase-Δ382C

Electron density corresponding to acarbose was observed only in domain A of each protomer. Acarbose spans subsites −1 to +3 in the pocket-shaped active site formed solely by domain A (Fig. 3, *E*, *F*, S10 and S11). The valienamine unit of acarbose occupied subsite −1 at the bottom of the pocket, and the 6-deoxy-d-glucose and the two d-glucose units at the reducing-end side bound to the rim of the active pocket, forming subsites +1, +2, and +3. In subsite −1, the valienamine unit had numerous potential hydrogen bonds and hydrophobic interactions, including O2 and O3 with main-chain carbonyl O of Ile669; O4 with Nε of Arg542 and Oδ1 of Asp543; O6 with Oδ2 of Asp543 and Oε2 of Glu874. O3 also formed a water (HOH404)-mediated hydrogen bond with Nε of Gln820. Ala529, Tyr535, and Trp540 formed a hydrophobic patch accommodating the C5, C6, and C7 atoms of the valienamine unit (equivalent to the C5, C6, and O5 of d-glucose, respectively). In subsite +1, O2 of the 6-deoxy-d-glucosyl residue possibly formed a water-mediated hydrogen bond with Oδ2 of Asp672 and Nη2 of Arg791, and O3 formed a hydrogen bond with Nη1 of Arg791 and carbonyl O of Trp670. A possible CH-π interaction was observed between Phe797 and the β-anomer face of the 6-deoxy-d-glucose unit at subsite +1, and between Trp803 and the β-anomer face of the d-glucose unit at subsite +2. Phe602 and Val603 interactions with the α-anomer face of d-glucose units were also observed at subsites +2 and + 3. A hydrogen bond was observed at subsite +2 between the main-chain carbonyl oxygen of Glu671 and the O2 atom of the d-glucose unit *via* a water molecule (HOH1404), whereas no hydrogen bond was observed at subsite +3. The acarbose exhibited five intramolecular hydrogen bonds: between the valienamine unit at subsite −1 and the 6-deoxy-d-glucose unit at subsite +1 (O2:O3), between the 6-deoxy-d-glucose unit and the d-glucose unit at subsite +2 (O2:O3), and between the d-glucose units at subsites +2 and + 3 (O2:O3, O5:O6, and O6:O6).

**Figure 4:**
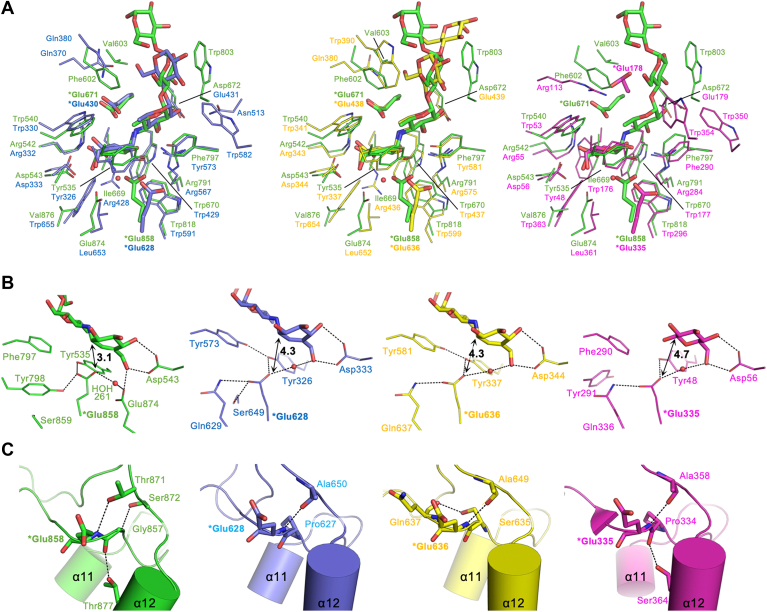
**Structural comparison of the active sites of GH15 enzymes.** Active site structures (*A*) and hydrogen-bond networks of side chains (*B*) and main chains (*C*) around the catalytic Glu residues (*asterisks*). The enzymes are color-coded as follows: *Green*, DDase with acarbose (PDB ID: 9JU0); *blue*, *Arthrobacter globiformis* I42 glucodextranase with acarbose (AgGD; PDB ID: 1ULV); *yellow*, *Thermoanaerobacterium thermosaccharolyticum* ATCC 7956 glucoamylase with acarbose (TtGA; PDB ID: 1LF9); magenta, *Kribbella flavida* NBRC14399 isomaltose glucohydrolase (KfIG; PDB ID: 5Z3A) superimposed with the β-d-glucose bound to its general base mutant (PDB ID: 5Z3F). The arrows with numerals indicate the distance (Å) between Oε2 of the catalytic Glu and the anomeric carbon equivalents of the ligands. *Red* spheres indicate putative nucleophilic water in the inverting enzymes. Potential hydrogen bonds are indicated by *dashed lines*. The α11 and α12 indicate the 11th and 12th α-helix of domain A, respectively. The main chain of the α11→α12 loop is shown as a cartoon model.

The acarbose-binding mode and the amino-acid residues involved in the acarbose binding in DDase are compared with GH15 inverting glucohydrolases (Fig. 4). Although the structures of the d-glucose units of acarbose binding at subsites +2 and + 3 varied among the enzymes, the structures of the valienamine and 6-deoxy-d-glucose units occupying subsites −1 and + 1 were well conserved across them (Fig. 4*A*). Glu671 and Glu858 of DDase correspond to the general acid and base catalysts of other GH15 enzymes, respectively, in the amino acid sequence, as described above. In the crystal structure, Oε2 of Glu671 is located 2.5 Å from the amine N atom of acarbose, which is equivalent to the glucosidic oxygen of natural substrates. The Glu671 position is almost identical to that of the general acid catalysts of other GH15 inverting hydrolases except KfIG (Fig. 4*A*) and is suitable as a proton donor. Glu858 is positioned on the β-anomer face side of the valienamine ring. Its Oε2 is 3.1 Å from C1 of the valienamine ring (anomeric carbon equivalent), approximately 1.2 to 1.6 Å closer than the corresponding distances in GH15 inverting glucoside hydrolases (Fig. 4*B*). Moreover, the Oε2 position is close to the nucleophilic water molecule in the inverting enzymes and is suitable for nucleophilic attack. The carboxy group of Glu858 possibly forms hydrogen bonds with Tyr535, Tyr798, and HOH261, which is hydrogen bonded with HOH404 and Glu874.

### Characterization of Glu671 and Glu858 mutant enzymes

Glu671 and Glu858 of DDase were mutated using Δ382C as the parent enzyme, and their functions were analyzed. Kinetic parameters of E671Q toward G4 and α-d-glucosyl fluoride (α-GF) were determined by measuring aglycon-releasing velocities (G3 and fluoride ion, respectively) (Table 1). E671Q exhibited a drastic decrease in *k*_cat_ and *k*_cat_/*K*_m_ compared with those of its parent for both G4 and α-GF; however, the decrease was less pronounced for α-GF. Relative *k*_cat_ and *k*_cat_/*K*_m_ values of E671Q compared to those of the parent enzyme were 1.0 × 10^−5^ and 6.4 × 10^−6^ for G4, while those for α-GF were 1.7 × 10^−3^ and 4.5 × 10^−2^, respectively. The less pronounced decrease in *k*_cat_ and *k*_cat_/*K*_m_ for α-GF, which possesses a good leaving group, suggests that Glu671 is the proton-donor catalyst, as indicated in GH15 glucoamylase (46) and other retaining glycosidases (24, 47, 48).

Glycosynthase reactions in the Glu858 mutants were examined. The glycosynthase reaction is a glycoside synthesis reaction using inactive nucleophile mutants, in which the nucleophile is replaced by a small residue, and glycosyl fluoride with the opposite anomeric configuration is used as a donor substrate (49). Four mutant enzymes, E858Q/A/C/S, exhibited no detectable activity on 15 mM G4 (<5.0 × 10^−3^ s^−1^) and 50 mM isomaltotriose (IG3) (<2.8 × 10^−4^ s^−1^), indicating that the mutations resulted in a decrease in activity to < 6.2 × 10^−5^-fold and < 1.4 × 10^−4^-fold those of the parent enzyme, respectively. However, isomaltotetraose (IG4) was produced from β-d-glucosyl fluoride (β-GF) and IG3 by E858A and E858S at the initial rates of 1.6 × 10^−2^ s^−1^ and 8.8 × 10^−2^ s^−1^, respectively. Isomaltose (IG2) was not detected in any reactions. The production of IG4 was not detected without β-GF by any mutants (at a rate less than 2.8 × 10^−4^ s^−1^) (Table 2). The parent enzyme, Δ382C, produced IG2 and IG4 at almost the same rate regardless of the presence of β-GF, indicating that β-GF was not a substrate or inhibitor for Δ382C under these conditions. The occurrence of the glycosynthase reaction with E858A/S and β-GF suggests that Glu858 is the nucleophile catalyst in the parent DDase.

**Table 2:** Initial velocities of Glu858 mutant enzymes

Enzyme	Substrate: IG3		Substrate: IG3 and β-GF	
	*v*_IG2_ (s^−1^)	*v*_IG4_ (s^−1^)	*v*_IG2_ (s^−1^)	*v*_IG4_ (s^−1^)
Δ382C	2.0 ± 0.0	1.8 ± 0.0	1.8 ± 0.0	1.8 ± 0.0
E858A	n.d.	n.d.	n.d.	0.016 ± 0.002
E858C	n.d.	n.d.	n.d.	n.d.
E858Q	n.d.	n.d.	n.d.	n.d.
E858S	n.d.	n.d.	n.d.	0.088 ± 0.024

*v*_IG2_ and *v*_IG4_: rates producing IG2 and IG4, respectively, from 50 mM IG3 and 0 or 12.5 mM β-GF. The values were expressed as mean ± standard deviation of triplicate measurements.

n.d.: not detected (less than 2.8 × 10^−4^ s^−1^).

## Discussion

DDase is a transglucosidase that produces dextran from dextrin through successive transglucosylations. This study analyzed the DDase structure, including determining the two catalytic residues, through structural and biochemical analyses. Surprisingly, the structure of domains N2 and A, including the connecting HLH motif, of DDase was similar to that of bacterial GH15 glucohydrolases. The pseudo-tetrasaccharide acarbose was bound to the active pocket in a manner almost identical to that of GH15 inverting glucohydrolases (Fig. 4*A*). Particularly, residues at subsite −1 are well conserved, including two catalytic residues, Glu671 and Glu858, which correspond to general acid and general base catalysts of GH15 inverting glucohydrolases, respectively. Glu671 is located at the same position as the general acid catalyst for glucohydrolases. Gln substitution for Glu671 caused a drastic decrease in rates for both α-GF and G4, but *k*_cat_ and *k*_cat_/*K*_m_ were 18- and 120-fold higher for α-GF than for G4, respectively, while those were 0.11- and 0.017-fold in the parent enzyme Δ382C (Table 1). This suggests that Glu671 is a proton donor, the mutation of which affects substrates harboring a poor-leaving group more considerably because they require more assistance from the general acid catalyst to be released (50, 51).

The other possible catalytic residue, Glu858, corresponds to the general base catalysts of GH15 glucohydrolases in the primary structures (Fig. 1), but its three-dimensional arrangement is subtly different from those of this family of enzymes. Glu858 of DDase is positioned 1.2 to 1.6 Å closer to the anomeric carbon of the substrate in subsite −1, compared to the inverting glucoside hydrolases, and Oε2 of Glu858 is located at an almost identical position to the substrate water in the inverting glucohydrolases (Fig. 4, *A* and *B*). In the inverting enzymes, the substrate water molecules are in a hydrogen-bonding network with their general base residues and a conserved Asp residue (Asp333 of AgGD, Asp344 of TtGA, and Asp56 of KfIG) *via* O4 and O6 of the valienamine or d-glucose moiety in subsite −1 (Fig. 4*B*). The hydrogen bond network is possibly invariant to adequately place the water molecule for nucleophilic attack and adjust the p*K*_a_ of the general base catalysts. In DDase, the equivalent Asp543 is located identically to form hydrogen bonds with O4 and O6 of the valienamine unit. Although no direct hydrogen bond is possible between O6 and Oε2 of Glu858, they are involved in another hydrogen bond network involving Glu874 and another water (HOH261 in Mol A) instead (Figs. 3*F* and 4*B*). The structural prediction that Glu858 serves as a catalytic nucleophile was supported by the glycosynthase reaction. E858A/S used the wrong-anomer substrate β-GF as the d-glucosyl donor for transglucosylation, whereas the E858Q variant and its parent Δ382C did not. The smaller side chain of the mutant, particularly Ser, provides space and better interaction for the fluorine atom of β-GF, as observed in E358S of *Agrobacterium* sp. β-glucosidase (52) and E197S of *Humicola insolens* endoglucanase Cel7B (53).

It is particularly intriguing that the acid/base and nucleophile catalytic residues of DDase are well conserved in sequence with the general acid and general base residues of GH15 inverting enzymes. This is distinct from the anomer-inverting and -retaining enzymes in GH23 and GH97, which share only one catalytic residue but differ in the other. In GH23, the two types of enzymes conserve Glu residue as a general acid or acid/base catalyst, but not the other catalytic residue: inverting goose-type lysozymes possess two Asp residues as a general base, whereas retaining lytic transglycosylases lack the corresponding Asp residues (54, 55). Lytic transglycosylases possibly use the acetoamide group of substrate as a catalytic nucleophile (substrate-assisted catalysis). In GH97, both inverting α-glucoside hydrolases and retaining α-galactosidases possess a Glu residue in the β6→α6 loop of the catalytic (β/α)_8_-barrel domain, which acts as either a general acid or a general acid/base catalyst (56). However, the other catalytic residue differs in location: in inverting enzymes, two Glu residues in β3→α3 and β5→α5 loops serve as a general base, whereas in retaining enzymes, the Asp residue located in β4→α4 loop acts as catalytic nucleophile. As the two catalytic Glu residues of DDase are conserved in the primary structure of GH15 enzymes, including inverting enzymes, the reaction mechanism of GH15 enzymes cannot be discriminated based only on sequence comparison. The findings of this study highlight the potential pitfalls of inferring catalytic mechanisms solely from the conservation of catalytic residues in amino acid sequences. A comparable observation is made in GH31, where both α-glucoside hydrolases and lyases share two catalytic Asp residues in their sequences (57).

One possible factor determining the unique spatial arrangement of Glu858 in DDase is the α11→α12 loop structure (Fig. 4*C*). The main chains of Glu858 and the neighboring Gly857 of DDase potentially form hydrogen bonds with the side chains of Thr871 and Ser872 in α11→α12 loop and Thr877 on α-helix 12. The GH15 inverting glucohydrolases possess similar hydrogen bonds, however, with the main-chain carbonyl of Ala (Ala650 of AgGD, Ala649 of TtGA, and Ala358 of KfIG), which is conserved in region S5 of GH15 inverting hydrolases (Figs. 1 and 4*C*). At the Gly857 position in DDase, AgGD, and KfIG possess Pro (Pro627 and Pro334, respectively), and TtGA has Ser635, the side chain of which is involved in a different hydrogen bond in the local structure.

Even outside the α11→α12 loop, the carboxy group of Glu858 of DDase is also involved in a hydrogen bond network distinct from those of GH15 inverting enzymes (Fig. 4*B*). Potential hydrogen bonds were observed with Tyr535 in the α1→α2 loop and Tyr798 in the α9→α10 loop in DDase. The Tyr535 involvement is similar to that of GH15 glucohydrolases (Tyr326 of AgGD, Tyr337 of TtGA, and Tyr48 of KfIG). However, it binds to the other oxygen atom of the catalytic carboxy group of Glu858. The Glu858-neighboring Ser859 does not participate in direct hydrogen bonding with Glu858, unlike the corresponding Gln residues in the other GH15 enzymes (Gln629 of AgGD, Gln637 of TtGA, and Gln336 of KfIG). In contrast, it allows Tyr798 to form a direct hydrogen bond with Glu858 in DDase. KfIG has a corresponding Tyr (Tyr291) that is not suitably positioned for forming a hydrogen bond with the general base Glu335 due to the presence of Gln336. In AgGD and TtGA, the Tyr residues in the α1→α2 are hydrogen-bonded to Tyr573 and Tyr581 in the α9→α10 loops. However, the corresponding residue in the α9→α10 loop is Phe in DDase and KfIG, preventing this equivalent hydrogen bond formation.

In addition to the local structure, the domain N1 potential involvement is discussed. Domain N1 of DDase, which is essential for catalytic activity, as indicated in the truncation experiments, it resembles CBM35 of various glycoside hydrolases, including GH66 CITase (PDB ID: 3WNK), GH2 exo-β-d-glucosaminidase (PDB ID: 2VZQ), and GH30 glucuronoxylan-specific xylanase (PDB ID: 4QAW). However, the domain N1 of DDase does not contain sugar-binding residues. Acarbose and any acarbose-derived compounds were not found outside the active pocket in the determined structure of the DDase-acarbose complex. This implies that domain N1 is not directly involved in sugar binding, although the possibility of its binding to a long-chain substrate or product could not be completely excluded. However, domain N1 is essential for forming the dimeric state and is presumably necessary for the appropriate structure of the active site of DDase. Within each protomer, the Glu858-containing α11→α12 loop of domain A directly contacts the β7→β8 and β10→α1 loops of the N1 domain and the loop connecting domains N1 and N2 (Fig. 3, *C* and *D*). Moreover, the α9→α10 loop of domain A, which is involved in direct hydrogen bond to the nucleophile catalyst (between Tyr798 and Glu858) and forms a part of the substrate binding site, directly contacts the protruding β4→β5 loop (Asn40, Ala41) of domain N1 of the other protomer in the dimeric state *via* hydrogen bonds (Fig. 3*D*). Therefore, the domain N1 interactions are presumably necessary for the correct positioning of the catalytic residues and substrate binding *via* maintaining the appropriate structure of the α9→α10 loop in DDase.

**Figure 5:**
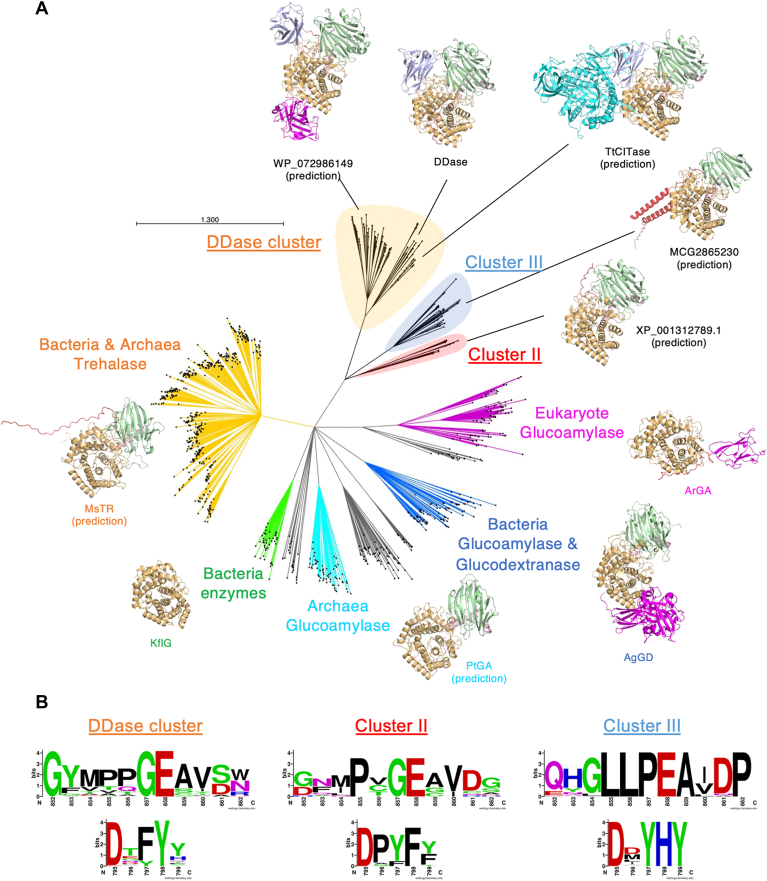
**Phylogenetic analysis of GH15 and related proteins.***A*, phylogenetic tree of GH15 and DDase-related enzymes and their structural information. The structures predicted using AlphaFold3 are indicated as (prediction). The enzyme abbreviations are as follows: DDase, *Gluconobacter oxydans* dextran dextrinase; ArGA, *Amorphotheca resinae* glucoamylase; AgGD, *Arthrobacter globiformis* glucodextranase; PtGA, *Picrophilus torridus* glucoamylase; KfIG, *Kribbella flavida* isomaltose glucohydrolase; MsTR, *Mycolicibacterium smegmatis* trehalase. *B*, sequence conservation around the Gly857-Glu858 dyad (in Region S5) and Phe797-Tyr798 dyad (in Region S4) of DDase within three-cluster proteins. Numbers are of DDase.

Phylogenetic analysis of the GH15 proteins indicated that DDase belongs to a cluster distinct from that of known inverting enzymes (Fig. 5). This cluster comprises DDase-like proteins, including TtCITase. These proteins share a Phe-Tyr dyad (in the conserved region S4) in the α9→α10 loop and a Gly-Glu dyad (in region S5) in the α11→α12 loop of domain A, which correspond to Phe797-Tyr798 and Gly857-Glu858 of DDase, respectively. In contrast, proteins belonging to clusters II and III, which are phylogenetically close to DDase, possess only the Gly-Glu dyad (cluster II) or lack both dyads (cluster III). Thus, the DDase cluster is most likely composed of retaining enzymes, whereas enzymes in clusters II and III are not expected to be anomer-retaining. Furthermore, the N-terminal CBM35-like N1 domain is found in almost half of the DDase cluster enzymes, yet not in other GH15 enzymes. Gene fusion and fission are believed to be the key mechanisms in protein evolution (58). An ancestral GH15 anomer-retaining enzyme might have acquired a CBM35-like N1 domain and evolved collaboratively with the catalytic domain and the N1 domain to enable the enzymes to catalyze the transglucosylation *via* altering the proximal and distal spatial arrangement of catalytic residues in the α11→α12 loop. Since some DDase-like enzymes, including TtCITase, are fusion proteins with GH66 enzymes, the CBM35-like N1 domain might have been derived from a GH66 protein.

Regarding the substrate specificity, DDase acts on both α-(1→4)-linked MOS and α-(1→6)-linked IMO. Docking analysis using AutoDock Vina suggested potential binding modes of G2 and IG2 as shown in Fig. S12, alongside alternative structures in which the ligands bound outside the active pocket of DDase. In the G2 binding structure, the molecule occupied subsites −1 and + 1 in the same mode as acarbose so that all the interactions at subsite +1 with Trp670, Asp672, Arg791, and Phe797 remained unchanged. The absence of hydrogen bonding at the O6 of the d-glucose unit at subsite +1 was confirmed, which might allow DDase to tolerate d-xylose as an acceptor substrate at subsite +1 (12). In the IG2 binding structure, the binding mode at subsite −1 was the same as that of G2. As anticipated, the reducing-end d-glucose unit was positioned slightly differently at subsite +1 compared to its position in the G2 binding mode. The binding appeared to be maintained through interactions with the residues involved in maltose binding; however, the binding counterparts shifted from O2 and O3 of G2 to O3 and O4 of IG2, respectively. Although the residues involved in binding MOS and IMO at subsites −1 and + 1 are almost conserved in the GH15 enzymes including DDase, different residues are likely responsible for forming subsites +2 and + 3 or around (Fig. 4*A*). As previously pointed out (27, 59), α-(1→4)-linkage-specific glucoamylases share Trp in the α3→α4 loop (Trp390 of TtGA) to form subsites +2 and + 3 for MOSs, and α-(1→6)-linkage-specific AgGD and KfIG possess spatially conserved Trp582 and Trp350, respectively, on the other side (Fig. 4*A*). The arrangement of these residues likely determines their substrate specificity. In DDase, the corresponding residues Phe602–Val603 and Trp803 may play important roles in binding MOS and IMO at subsites +2 and +3.

In conclusion, the present study demonstrated the structural similarity between DDase and GH15 inverting glucohydrolases. Glu671 in the α5→α6 loop is the acid/base catalyst, located at the same position as the general acid catalyst of the anomer-inverting enzymes. The nucleophile catalyst is Glu858 in the α11→α12 loop, which is positioned slightly differently and involved in a distinct hydrogen bonding network compared to the inverting enzymes, although it is equivalent to the general base catalyst in the sequence. The biochemical analyses of the catalytic residue mutants with α-GF and β-GF support the proposed catalytic roles of these residues. The structure suggests that the spatial arrangement of nucleophile catalyst Glu858 involves interaction with the α9→α10 loop of domain A, and other domains, including domain N1 of the other protomer in the dimeric state. Domain N1 is exclusively found in approximately half of the enzymes within DDase cluster and is absent from other GH15 inverting enzymes, raising the possibility that the ancestral protein might have acquired domain N1 during the evolutionary process of adopting the retaining mechanism.

## Experimental procedures

### Materials

A series of MOSs, IMOs, and acarbose were purchased from Nihon Shokuhin Kako (Tokyo, Japan), Tokyo Chemical Industry, and Carbosynth, respectively. α-GF and β-GF were synthesized according to previous publications (60, 61).

### N-terminal and internal amino acid sequence analysis of DDase

Extracellular DDase from *G. oxydans* ATCC 11894 was purified as described previously (39). The purified enzyme was transferred to a polyvinylidene difluoride membrane from an SDS-PAGE gel *via* electroblotting using a semi-dry blotting apparatus. The N-terminal amino acid sequence was determined by Edman degradation using a Procise 491 analyzer (Thermo Fisher Scientific). To determine partial internal sequences, tryptic peptides of DDase were prepared by incubating 97 μg of DDase with 1 μg of trypsin (Sigma-Aldrich, Merck, Darmstadt, Germany) in 200 μl of 0.1 M Tris-HCl buffer (pH 7.0) containing 2 M urea at 37 °C for 24 h. Subsequently, 3.2 μl of 2-mercaptoethanol was added, and the sample was kept at room temperature under a nitrogen atmosphere for 16 h. Then, the pH was adjusted to 4.5 with acetic acid, and 4.8 μl of 4-vinylpyridine was added, followed by incubation for an additional 2 h. The resulting peptides were separated by HPLC under the following conditions: column, Capcell Pak C_18_ UG120 (4.6 mm I.D. × 150 mm; Shiseido, Tokyo, Japan); column temperature, 50 °C; flow rate, 1 ml/min; elution gradient, 0 to 50% acetonitrile in 0.1% trifluoroacetic acid; detection, absorbance at 214 nm. Amino acid sequences of the separated peptides were analyzed as described above.

### Cloning of the DDase gene

*G. oxydans* genomic DNA was prepared from bacterial cells cultured in 100 ml of medium consisting of 5 g/L Bacto yeast extract (Thermo Fischer Scientific) and 25 g/L mannitol (Nacalai Tesque, Kyoto, Japan) at 30 °C for 48 h. Nucleic acids were extracted from bacterial cells using 10 g/L SDS after treatment with lysozyme (Sigma-Aldrich) and Proteinase K (Life Technologies) in 10 mM Tris-HCl buffer (pH 8.0) containing 20 mM EDTA. Genomic DNA was subsequently purified by phenol-chloroform extraction, followed by RNase A treatment and ethanol precipitation.

A partial DDase gene was amplified from the genomic DNA by PCR using degenerate primers (Table S3), then inserted into the pGEM-T vector (Promega) for cloning. The amplified DNA was labeled with the Alkphos Direct Labeling Reagent (GE Healthcare) and used as a probe for screening. A partial *G. oxydans* genomic DNA library, containing 6.6 to 23 kbp fragments of NcoI-digested genomic DNA was constructed in a modified pBluescript II SK vector (Agilent Technologies). In this modified vector, a NcoI site had been introduced between the XhoI and EcoRI sites using annealed oligonucleotides with the sequences 5′-TCGAGCCATGGG-3′ and 5′-AATTCCCATGGC-3′. Colony hybridization using the labeled probe was used to obtain the 5ʹ-region of the DDase gene. Its 3′-region was amplified from EcoRI, SalI, and HindIII digests using an *in vitro* Cloning Kit (Takara Bio). All sequence analyses were performed using an ABI PRISM 310 Genetic Analyzer (Thermo Fisher Scientific).

### Construction of expression plasmids of DDase and its variants

The full-length DDase gene was amplified by PCR using primers DD_s9 and DD_a3 and inserted into the pCold I plasmid (Takara Bio) at the SacI and XbaI sites. For the truncation mutants, primers DD_s10–12 and DD_a4–8 were used. For other mutants: Δ422N/Δ382C, E671Q, E858A, E858C, E858Q, and E858S, the plasmid for Δ382C was mutated using a PrimeSTAR Max Mutagenesis kit (Takara Bio) and primers DD_s13–18 and DD_a9–14. All primer sequences are listed in Table S3. All the recombinant DDases contained 19 additional plasmid-derived residues, MNHKVHHHHHHIEGRHMEL, at the N-terminus of the full or indicated regions of DDase. These residues are not included in the amino acid residue numbers used in this study.

### Preparation of DDase variants

*E. coli* BL21-CodonPlus (DE3)-RIL transformants harboring the expression plasmid were cultured in LB broth containing 25 μg/ml ampicillin and 30 μg/ml chloramphenicol at 37 °C until the optical density at 600 nm (OD_600_) reached approximately 0.4 to 0.5. The culture was continued at 15 °C for 22 h with 0.5 mM isopropyl β-d-thiogalactopyranoside (Fujifilm Wako Pure Chemical). The cells were harvested by centrifugation, suspended in 50 mM HEPES-NaOH buffer (pH 7.0), and lysed by sonication. The supernatant obtained after centrifugation was subjected to Ni^2+^-affinity column chromatography (Chelating Sepharose Fast Flow; GE Healthcare). The column was equilibrated with 20 mM sodium phosphate buffer (pH 7.2) containing 0.5 M NaCl and washed with the same buffer containing 20 mM imidazole. The bound proteins were eluted by an increasing linear imidazole gradient (20–500 mM). The DDase-active fractions were pooled, dialyzed against 20 mM sodium acetate buffer (pH 5.3), and concentrated using centrifugal ultrafiltration filters (Vivaspin 20, 10 kDa molecular weight cut-off; Sartorius). All the purification steps were performed at 4 °C. The concentrations of the purified enzymes were determined using amino acid analysis after acid hydrolysis (6 N HCl, 110 °C, 24 h). The analysis was performed using a JLC-500/V amino acid analyzer (JEOL) equipped with a ninhydrin detection system.

### Standard enzyme assay

A reaction mixture (80 μl) containing enzyme (wild type, 0.00194 μM; Δ35N, 2.77 μM; Δ83N, 5.15 μM; Δ126N, 7.70 μM; Δ499C, 13.1 μM; Δ432C, 9.65 μM; Δ382C, 0.00335 μM; Δ255C, 0.00438 μM; Δ130C, 0.00239 μM; Δ422N/Δ382C, 5.85 μM), 15 mM G4, 30 mM sodium acetate buffer (pH 4.2), and 0.2 mg/ml bovine serum albumin (BSA; Nacalai Tesque) was incubated at 35 °C for 3 to 30 min. Aliquots (15 μl) were sampled and heated at 100 °C for 3 min to terminate the reaction. The G3 concentration was quantified using high-performance anion exchange chromatography with pulsed amperometric detection (HPAEC-PAD).

For activity measurement, the reaction mixture (20 μl) consisting of 200 mM G2, 53 mM sodium acetate buffer (pH 4.2), 0.1 mg/ml BSA, and the enzyme was incubated at 35 °C for 10 min. The reaction was stopped by adding 40 μl of 2 M Tris-HCl (pH 7.0), and the liberated d-glucose was measured using the glucose oxidase-peroxidase method (62) with the Glucose C-II-Test Wako kit (Fujifilm Wako Pure Chemicals). One unit of DDase was defined as the amount of enzyme producing 1 μmol d-glucose per minute under these conditions.

For producing megalosaccharides and polysaccharides, the enzyme (0.1 U) was incubated with 100 mg/ml maltohexaose- and maltoheptaose-rich syrup (Fujioligo G67; Nihon Shokuhin Kako) in 50 mM sodium acetate buffer (pH 4.2) at 35 °C for 6 h. Polysaccharide and megalosaccharide fractionations by precipitation with 40 and 90% (v/v) methanol were performed according to a previous report (10).

### Effect of temperature and pH

To investigate the temperature effect, a reaction mixture of 259 nM DDase, 200 mM G2, and 33.3 mM sodium acetate buffer (pH 4.5) was kept at 26.5 to 60 °C. To examine the pH effect, a reaction mixture of 259 nM DDase, 200 mM G2, and 33.3 mM buffer, either sodium citrate buffer (pH 2.6–4.7), sodium acetate buffer (pH 4.0–6.0), or sodium phosphate buffer (pH 6.1–6.9), was incubated at 37 °C. Aliquots (20 μl) were sampled at 10, 20, and 30 min intervals and heated at 100 °C for 3 min to stop the reaction. The d-glucose concentration in these aliquots was quantified using the glucose oxidase-peroxidase method after adequate dilution with water.

To evaluate enzyme stability, 385 nM DDase was incubated in 50 mM sodium acetate buffer (pH 4.5) at temperatures ranging from 26.5 to 60 °C for 10 min to assess thermal stability. For pH stability assessment, the same amount of the enzyme was incubated in 50 mM buffers, either glycine-HCl buffer at pH 1.8 to 2.8, sodium citrate buffer at pH 2.4 to 4.2, sodium acetate buffer at pH 3.6 to 5.9, or sodium phosphate buffer at pH 6.0 to 7.2, at 4 °C for 24 h. Residual enzymatic activity was then assayed with 200 mM G2 at pH 4.5 and 37 °C. The stable regions were defined as the temperature and pH ranges exhibiting residual activity of more than 90% and 80%, respectively.

### HPAEC-PAD

HPAEC-PAD with a CarboPac PA1 column (4 × 250 mm) (Dionex, Sunnyvale, CA, USA) was used. An isocratic mobile phase of 640 mM and 320 mM NaOH was applied to separate a series of MOSs and IMOs, respectively, at a 0.8 ml/min flow rate. d-Glucitol (100 μM; Nacalai Tesque) was used as an internal standard.

### Kinetic analysis

The substrate concentration effect on reaction rates was analyzed using a series of MOS from G2 to maltoheptaose (G7) and α-GF. A reaction mixture (80 μl) containing enzyme, substrate, 30 mM sodium acetate buffer (pH 4.2), and 0.2 mg/ml BSA was incubated at 35 °C for 3 to 30 min. Aliquots (15 μl) were sampled and heated at 100 °C for 3 min to terminate the reaction. Substrate concentrations ranged from 20 to 200 mM for G2, 5 to 120 mM for G3–G7, or 1 to 100 mM for α-GF. Enzyme concentrations were as follows: 388 nM (G2), 1.94 nM (G3), and 0.970 nM (G4–G7) for the wild-type enzyme; 670 nM (G2) and 3.35 nM (G3–G7, α-GF) for the Δ382C variant; and 7.45 μM (G4, α-GF) for the E671Q variant. In the reactions of E671Q and Δ382C with G4 and α-GF, a 130 mM buffer was used, and the reaction time was extended to 120 min.

Reaction rates were determined by quantifying the released aglycons using HPAEC-PAD (d-glucose and MOS) or a colorimetric method (fluoride ion) employing a fluoride ion-specific dye (63), Alfusone (Dojindo). The kinetic parameters (*k*_cat_ and *K*_m_) were determined by fitting the experimental data to the Michaelis–Menten equation using KaleidaGraph 3.6 J software (Synergy Software, Reading, PA, USA).

### Glycosynthase reaction

Enzymes (0.108 μM Δ382C, 0.87–11.3 μM E858A/C/Q/S) were incubated with 50 mM IG3, 0 or 12.5 mM β-GF, 0.2 mg/ml BSA, and 130 mM sodium acetate buffer (pH 4.2) at 35 °C for 3 to 120 min. Aliquots (15 μl) were sampled, and the reaction was stopped as described above. The produced IG2 and IG4 were quantified by HPAEC-PAD.

### X-ray crystal structure analysis

The Δ382C variant purified by Ni^2+^-affinity column chromatography was further purified using a Q-Sepharose column (GE Healthcare), pre-equilibrated with 20 mM sodium acetate buffer (pH 5.3). The column was washed with 100 mM sodium acetate buffer (pH 5.3), followed by elution with a linear gradient of NaCl (0–100 mM) and pH (5.3–4.0) in 100 mM sodium acetate buffer. The active fractions were pooled, dialyzed against 20 mM glycine-HCl buffer (pH 2.5), and concentrated by ultrafiltration.

Protein crystallization was performed *via* the hanging drop vapor diffusion method at 20 °C for approximately 1 year. Several Δ382C variant crystals were obtained from a droplet containing 5 μl of protein solution (12 mg/ml), 1 μl of 1 mM acarbose, and 1 μl of reservoir solution [10% (w/v) polyethylene glycol 6000, 6% (v/v) ethylene glycol, and 0.1 M sodium acetate buffer (pH 3.5)]. The crystals were then soaked in a cryoprotectant solution [10% (w/v) polyethylene glycol 6000, 6% (v/v) ethylene glycol, 0.1 M sodium acetate buffer (pH 3.5), 20% (v/v) glycerol, and 1 mM acarbose] and flash-frozen in liquid nitrogen.

For S-SAD phasing, a nylon loop and the solvent surrounding the crystal were ablated using a deep-UV laser at a wavelength of 193 nm under a nitrogen gas stream at 100 K (64). The 55 datasets from the crystal were collected at beamline BL-1A of the Photon Factory (Tsukuba, Japan) at a wavelength of 2.7 Å under a helium atmosphere. A high-resolution dataset was collected from a single crystal in a cryoprotectant at beamline BL26B2 of Spring-8 (Hyogo, Japan) at a wavelength of 1.0 Å under a nitrogen gas stream at 100 K. The diffraction datasets were processed using XDS and merged with XSCALE (65). The phase problem was solved using S-SAD. Sixty-six of the 80 sulfur sites were found (occupancy > 0.3, Fig. S7) in SHELX (66) and HKL2MAP (67). Automatic model building was performed using CRANK2 (68) within CCP4. Eight hundred ninety-eight out of 921 residues in each protomer were built and subsequently used as the search model for the molecular replacement in the high-resolution structural analysis using AutoMR within PHENIX (69). The refinement process was carried out using Refmac5 (70) within CCP4 and phenix.refine (71) in conjunction with interactive model fitting and rebuilding based on 2m*F*o-D*F*c and m*F*o-D*F*c electron densities using COOT (72). The data collection and refinement statistics are summarized in Table S2.

The PDBQTfiles of G2, IG2, and DDase-Δ382C (Glu671 was treated as flexible) were prepared using AutoDockTools (73), and the docking experiments were performed using AutoDock Vina (74).

Graphical representations were prepared using the PyMOL Molecular Graphics System (version 2.0; Schrödinger, LLC). A polder omit map was generated using Phenix.polder (75).

### Phylogenetic analysis

The amino acid sequences of all GH15 sequences deposited in NCBI, and the top 250 sequences identified by a BLAST search, conducted against the refseq_protein database using DDase as the query sequence, were retrieved. Sequences shorter than 350 residues were excluded, and the remaining sequences were clustered using DIAMOND Deep-Clust (76) with default parameters. The resulting 1088 sequences, including those of characterized GH15 enzymes, were aligned using MAFFT, applying the FFT-NS-i method (77). A neighbor-joining phylogenetic tree was constructed with 200 bootstrap replicates and depicted with a bootstrap threshold > 70% using CLC Genomics Workbench version 23.0.4. Sequences within the three clusters were aligned using MUSCLE (78), and their sequence logos were generated using WebLogo (79).

## Data availability

The crystal structure of DDase Δ382C bound with acarbose has been deposited into the Protein Data Bank (PDB) with accession ID 9JU0.

## Supporting information

This article contains supporting information including supplemental Tables S1–S3 and Figs. S1–S12.

## Conflict of interest

The authors declare that they have no conflicts of interest related to the content of this study.
