# Supplementary material for: Structural basis of transglucosylation in dextran dextrinase, a homolog of anomer-inverting GH15 glucoside hydrolases
Source: J Biol Chem. 2025 Jul 30;301(9):110541. doi: 10.1016/j.jbc.2025.110541 (PMC12446779; doi:10.1016/j.jbc.2025.110541)
Supplement: Supporting Figures [file mmc1.pdf]

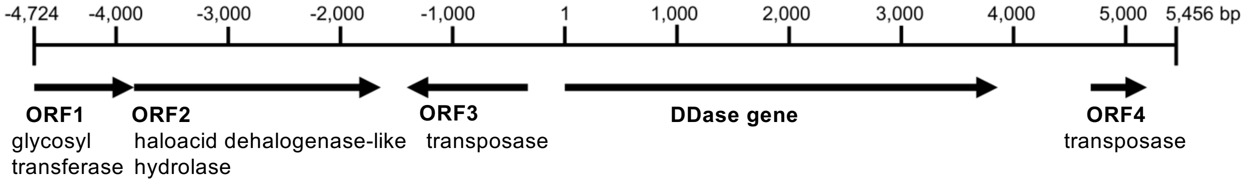

Figure S1. Linear map of a 10,180-bp genome fragment containing the DDase gene of *Gluconobacter oxydans* ATCC 11894.
The genes encoding DDase and putative ORFs are indicated by arrows. The arrows reflect the directions of transcription. Predicted functions of the ORFs are shown as the top hit from BLAST search.

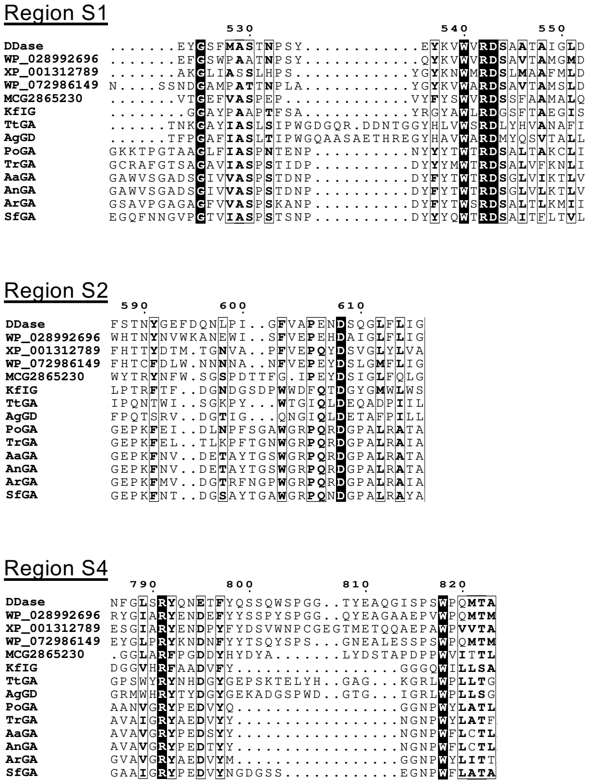

Figure S2. Multiple sequence and structure alignment of regions S1, S2, and S4 conserved in GH15 enzymes.

Multiple sequence and structural alignment was constructed and visualized as in Figure 1.

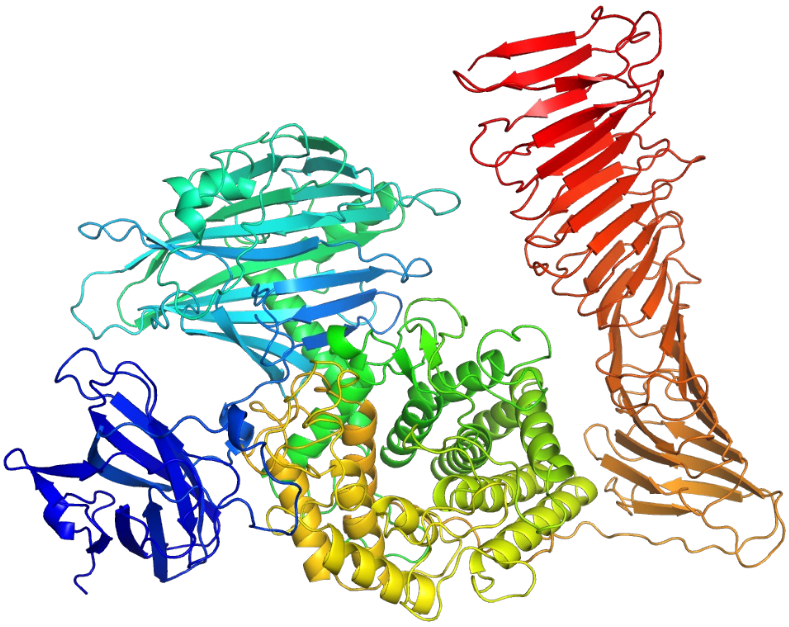

Figure S3. Predicted structure of intact DDase.
Prediction of the overall structure of DDase was performed with AlphaFold3. The predicted structure is drawn as a cartoon model with color spectrum from N (blue) to C (red) terminus using PyMOL.

Supporting Information Figures (Tagami, *et al.*)

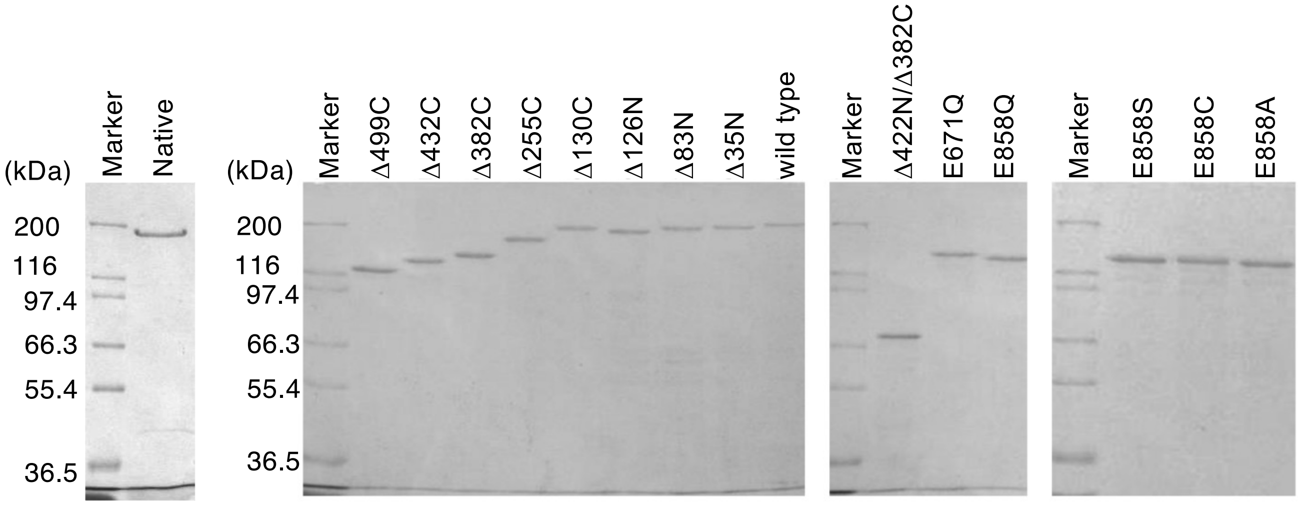

Figure S4. SDS-PAGE analysis of the purified native and recombinant-DDase derivatives.

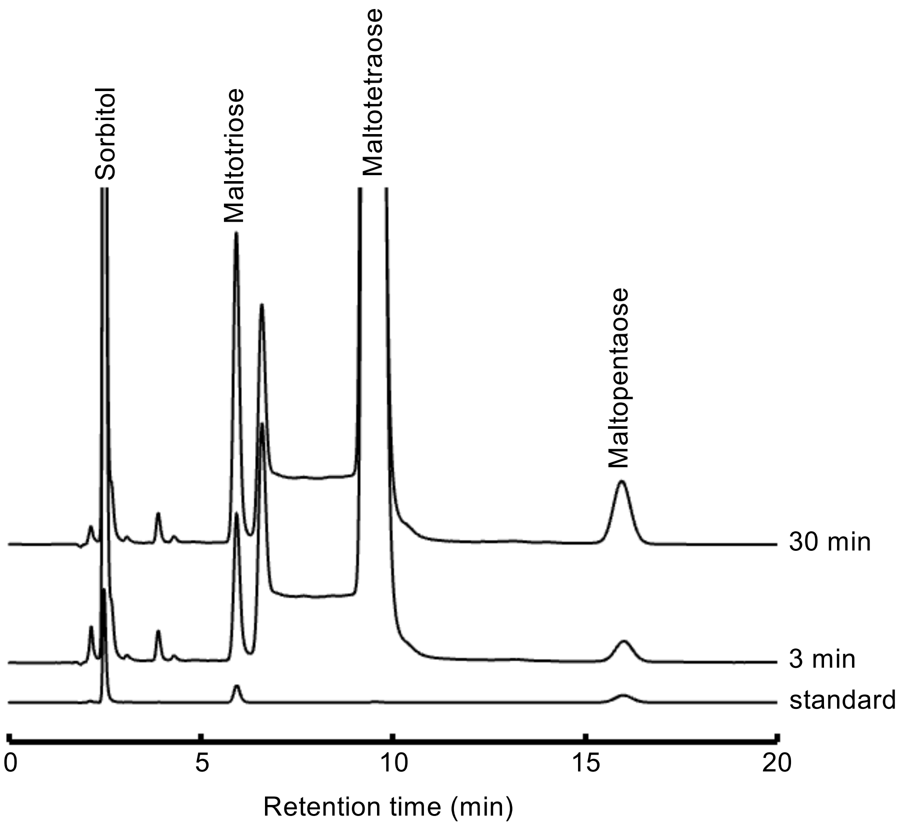

Figure S5. HPAEC-PAD analysis of the reaction products of wild-type DDase with maltotetraose (15 mM).

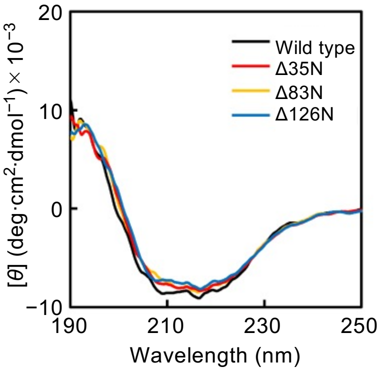

Figure S6. CD spectra of the N-terminal truncation variants of DDase.
All measurements were performed using protein samples at concentrations ranging from 8.6 to 14 μg/mL in 0.2 mM sodium acetate buffer (pH 5.3) at 22°C. Ultraviolet CD spectra (190–250 nm) were recorded using a JASCO 715 spectropolarimeter (Jasco, Tokyo, Japan) using 1-cm path length cuvettes. Two spectra were collected, averaged, and corrected by subtracting the blank buffer spectrum.

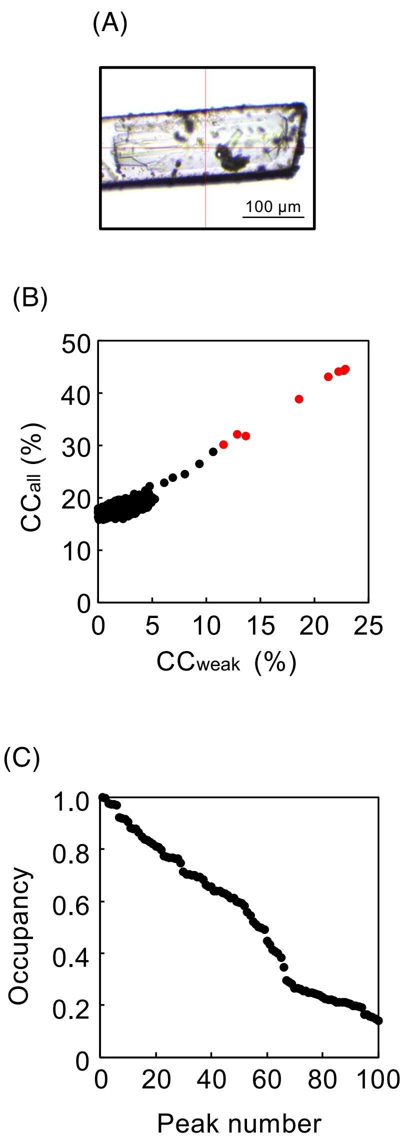

Figure S7. Sulfur-single-wavelength anomalous dispersion phasing for the crystal structure analysis of DDase-Δ382C.

(A) The co-crystal of DDase-Δ382C with acarbose mounted a nylon loop and shaped with a UV laser of wavelength 193 nm. The central pink square indicates the beam size (13 × 13 μm^2). (B) CC_{all} versus CC_{weak} plots and (C) occupancy versus peak number plots from the SHELXD solution for the 360° × 55 datasets at 2.7 Å wavelength. The red plots indicate CC_{all} >30%.

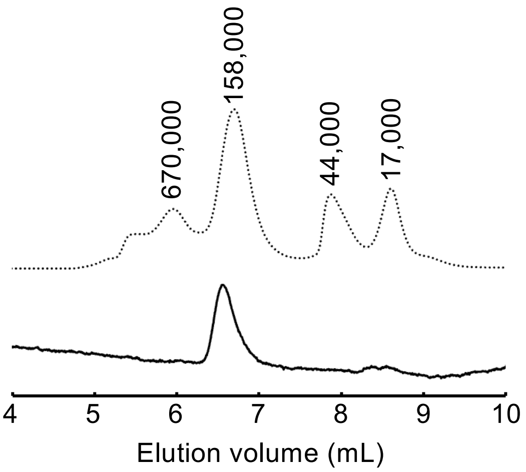

Figure S8. Gel-filtration column chromatography.
Gel-filtration column chromatography was performed using COSMOSIL 5-Diol-300-II column (7.5 mm I.D.×300 mm) and 100 mM sodium acetate buffer (pH 4.2) as eluant. Proteins were monitored by A_{280}. Dashed line, standard (The numerals indicate molecular weight of the proteins.); solid line, DDase Δ382C.

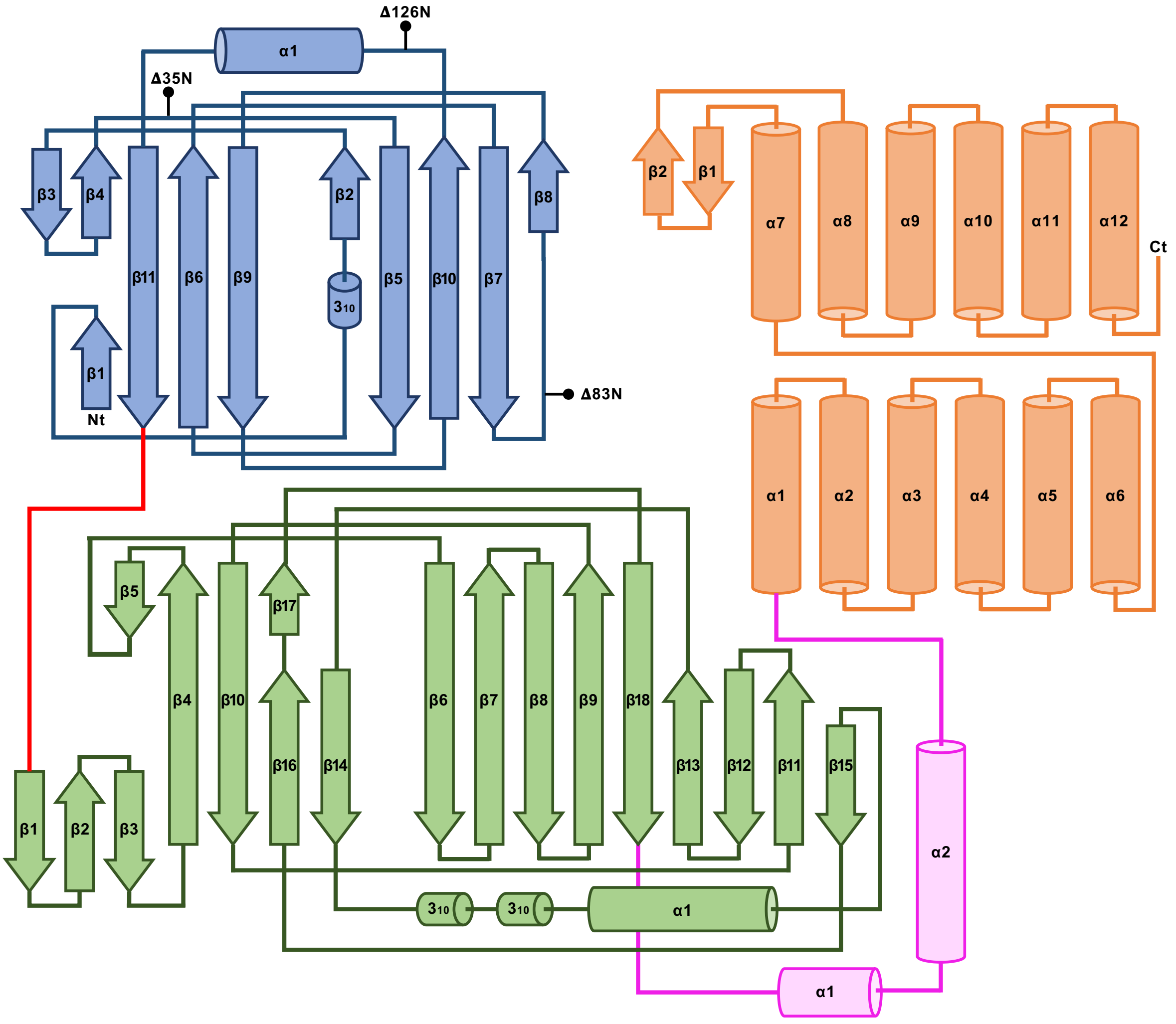

Figure S9. Topology of DDase-Δ382C.
The arrow, cylinder, and small cylinder indicate β-strand, α-helix, and 3_{10} helix, respectively. The color coordinate is the same as Figure 3B. Nt, N-terminal end, Ct, C-terminal end. Δ35N, Δ83N, and Δ126N indicate the theoretical N-termini of the N-terminal truncation mutants.

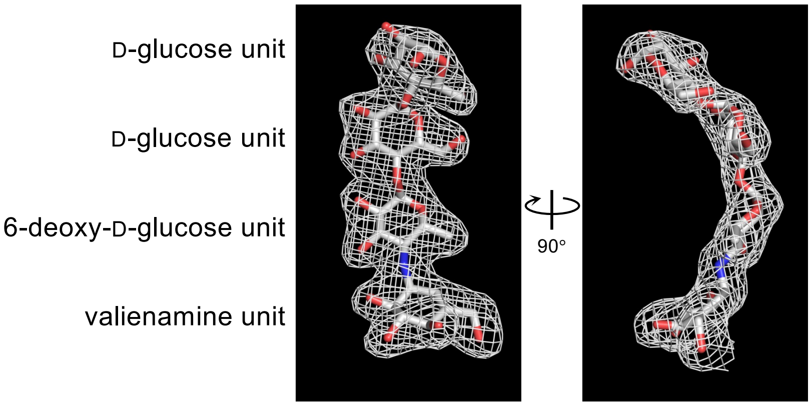

Figure S10. Polder omit map corresponding to acarbose contoured at 4 σ observed in domain A of Mol A.

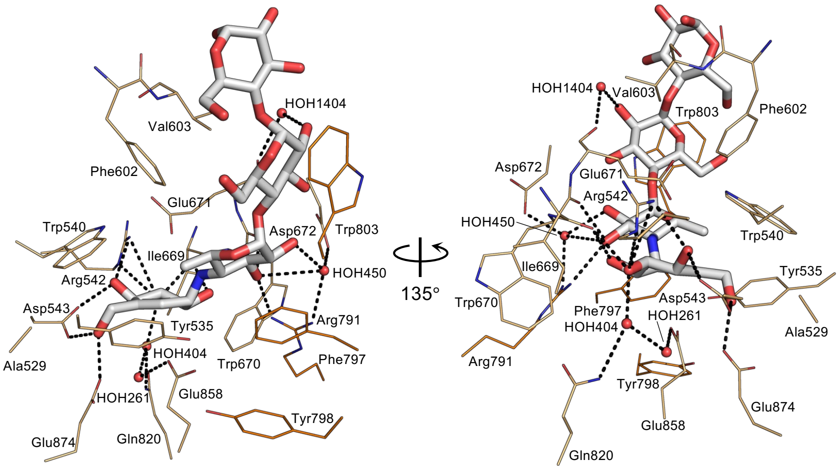

Figure S11. Active-site structure of Mol A with two angles.
Hydrogen bonds and water molecules are shown as dashed lines and red spheres, respectively.

Maltose

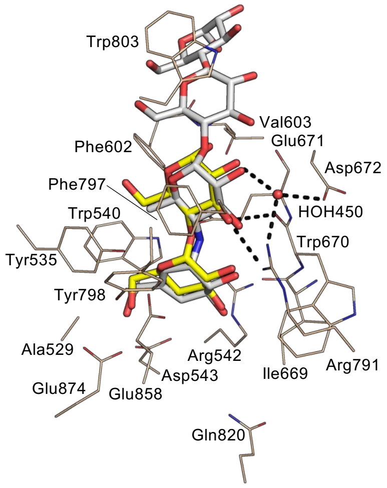

Isomaltose

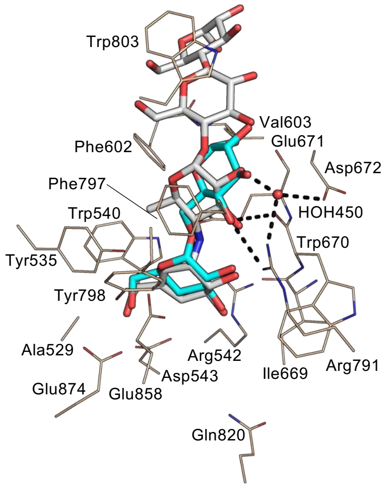

Figure S12. Docking simulations of maltose and isomaltose with DDase.
One of the binding modes of maltose (yellow; affinity: −5.9 kcal/mol) and isomaltose (cyan; affinity: −6.2 kcal/mol) are superimposed to the DDase structure bound with acarbose (white). The structures are shown as stereo diagram. Dashed lines and red sphere indicate possible hydrogen bonds and water, respectively.
