# Supplementary material for: Structural basis of transglucosylation in dextran dextrinase, a homolog of anomer-inverting GH15 glucoside hydrolases
Source: J Biol Chem. 2025 Jul 30;301(9):110541. doi: 10.1016/j.jbc.2025.110541 (PMC12446779; doi:10.1016/j.jbc.2025.110541)
Supplement: Supporting Tables [file mmc2.pdf]

Supporting Information Tables (Tagami, *et al.*)

Table S1. N-terminal amino acid sequences of native DDase and its tryptic peptides determined by Edman degradation.

Peptide	Sequence	Position
Intact	ADNSDEQFVA	2–11
#1	AEGLNAASQLASAMGEGNQA	685–704
#2	XXXLNLGTDGQK	1099–1110
#3	TGINPGEVSSTTXDP	294–310
#4	NVTPGQQAETYSPYFK	214–229
#5	ASVYLK	102–107

The X indicates unidentified amino acids.

Table S2. Data collection and refinement statistics of the X-ray crystal structure analysis of DDase-Δ382C bound with acarbose.

	S-SAD	DDase-Δ382C+Acarbose
PDB ID	–	9JU0
Diffraction source	PF BL1A	SP8 BL26B2
Wavelength (Å)	2.7	1.000
Data processing		
Space group	$P2_12_12_1$	$P2_12_12_1$
Uni-cell parameters (Å)	117.0, 140.0, 265.0	118.3, 139.0, 264.5
Mosaicity (°)	–	0.139
Resolution range (Å)	49.67–3.10 (3.29–3.10)	49.11–2.50 (2.65–2.50)
Total No. of reflections	58102893 (9056183)	1141957 (182487)
No. of unique reflections	152497 (24507)	150716 (24036)
Multiplicity	381 (369)	7.6 (7.6)
Completeness (%)	100 (100)	99.9 (99.7)
Average $I/\sigma(I)$	63.03 (25.09)	8.64 (1.70)
Rmeas (%)	23.3 (54.9)	25.2 (120.2)
Rsym (%)	23.3 (54.9)	23.6 (119.4)
CC1/2	100 (99.8)	99.0 (71.3)
SigAno	1.685 (1.037)	0.767 (0.680)
Refinement		
R-work	–	0.192
R-free	–	0.218
Number of atoms	–	
Macromolecules	–	26697
Ligands	–	310
Solvent	–	1450
Protein residues	–	3577
RMS (bonds)	–	0.013
RMS (angles)	–	1.73
Ramachandran (%)		
Favored	–	97.64
Allowed	–	2.36
Outliers	–	0
Rotamer outliers (%)	–	0.84
Clash score	–	2.16

The parenthesized values are for the highest-resolution shell.

Table S3. Sequence of primers used in this study

Name	Sequence (5'→3')	Purpose
DD_s1	GCIGATAATWSIGATGAGCAGTTYGT	Degenerated sense primer to amplify the partial DDase gene
DD_a1	TCICCIGGGTTIATICCIGTNGC	Degenerated antisense primer to amplify the partial DDase gene
DD_s2	CCGATTACACGCTGCTTGGTGACGC	Sense primer to prepare the probe
DD_a2	TCATCGGTCGTCTGATTATTCAGTT	Antisense primer to prepare the probe
DD_s3	CGTCAGGCTCTGGTCAACTC	Sense primer in the first PCR with *Eco*RI digests of the *G. oxydans* genomic DNA
DD_s4	GGATCCAGGAAAATATTGGTAGC	Sense primer in the second PCR with the *Eco*RI digests
DD_s5	CGGTCGCCATGGTCCGCAATGAAGC	Sense primer in the first PCR with *Sal*I digests
DD_s6	TGGCAATGCCGGCGATACGAACGTT	Sense primer in the second PCR with the *Sal*I digests
DD_s7	GACGACGTCCGATGCCTATATCACG	Sense primer in the first PCR with *Hin*dIII digests
DD_s8	CTGGACGCGATCGACAATCAAGGCA	Sense primer for the second PCR with the *Hin*dIII digests
DD_s9	AAAAAGAGCTCATGGCTGACAACTCTGACGAG	Sense primer to prepare the expression plasmid of the wild type. *Sac*I site is underlined.
DD_a3	AAAAATCTAGATCAGGCGCCGACGATGTTCAT	Antisense primer to prepare the wild type. *Xba*I site is underlined.
DD_s10	CCGGTGAGCTCCCAGCCACGACCAACGCGGTT	Sense primer for Δ35N-expression plasmid. *Sac*I site is underlined.
DD_s11	GCCAGGAGCTCCCCGGGGTCGTCGAATTCGAC	Sense primer for Δ83N-expression plasmid. *Sac*I site is underlined.
DD_s12	CCAATGAGCTCACACCGGTGCCGGAGGATCAG	Sense primer for Δ126N-expression plasmid. *Sac*I site is underlined.
DD_a4	GTATATCTAGACGACTGGCTGGCGTTGATGGT	Antisense primer for Δ130C-expression plasmid. *Xba*I site is underlined.
DD_a5	GTCGTTCTAGAGGCCTGAATTTCCGTCAGAGG	Antisense primer for Δ255C-expression plasmid. *Xba*I site is underlined.

DD_a6	GCGGTTCTAGACTGCCCGGTAATCGCAGGCAT	Antisense primer for Δ382C-expression plasmid. *Xba*I site is underlined.
DD_a7	GGCGGTCTAGAGCCATTGTCATAGGACTGTTC	Antisense primer for Δ432C-expression plasmid. *Xba*I site is underlined.
DD_a8	AGACCTCTAGAGTCCTGCGTCAGCGCATTTTC	Antisense primer for Δ499C-expression plasmid. *Xba*I site is underlined.
DD_s13	GGAGCTCACCAGCCTGTCGCAGCTC	Sense primer for Δ422N/Δ382C-expression plasmid.
DD_a9	AGGCTGGTGAGCTCCATATGCCTACC	Antisense primer for Δ422N/Δ382C-expression plasmid.
DD_s14	ATCTGGCAGGACATGTACGGCTATCAT	Sense primer for E671Q-expression plasmid.
DD_a10	CATGTCCTGCCAGATTGAGTAGTCAGC	Antisense primer for E671Q-expression plasmid.
DD_s15	CCCGGTGCATCCTACGACTGGGCGCGT	Sense primer for E858A-expression plasmid.
DD_a11	GTAGGATGCACCGGGCGGAGTGCCGCC	Antisense primer for E858A-expression plasmid.
DD_s16	CCCGGTTGCTCCTACGACTGGGCGCGT	Sense primer for E858C-expression plasmid.
DD_a12	GTAGGAGCAACCGGGCGGAGTGCCGCC	Antisense primer for E858C-expression plasmid.
DD_s17	CCCGGTCAATCCTACGACTGGGCGCGT	Sense primer for E858Q-expression plasmid.
DD_a13	GTAGGATTGACCGGGCGGAGTGCCGCC	Antisense primer for E858Q-expression plasmid.
DD_s18	CCCGGTAGCTCCTACGACTGGGCGCGT	Sense primer for E858S-expression plasmid.
DD_a14	GTAGGAGCTACCGGGCGGAGTGCCGC	Antisense primer for E858S-expression plasmid.
